# Efficacy of Levothyroxine in Migraine Headaches in Children with Subclinical Hypothyroidism

**Published:** 2012

**Authors:** Mehrdad MIROULIAEI, Razieh FALLAH, Nasrollah BASHARDOOST, Mina PARTOVEE, Mahtab ORDOOEI

**Affiliations:** 1 Assistant Professor of Pediatric Endocrinology, Department of Pediatrics, Shahid Sadoughi University of Medical Sciences, Yazd, Iran; 2 Associate Professor of Pediatric Neurology, Department of Pediatrics, Shahid Sadoughi University of Medical Sciences, Yazd, Iran; 3 Professor of Epidemiology, Department of Biostatistics and Epidemiology, Ali-ebn-Abitaleb School of Medicine, Islamic Azad University, Yazd Branch, Yazd, Iran; 4 General Physician; 5 Assistant Professor of Pediatric Endocrinology, Department of Pediatrics, Shahid Sadoughi University of Medical Sciences, Yazd, Iran

**Keywords:** Headache, Migraine, Children, Hypothyroidism, Subclinical Hypothyroidism

## Abstract

**Objective:**

Hypothyroidism may be an exacerbating factor for primary headaches and migraine is one of the most common primary headaches in childhood. The purpose of this study was to evaluate the effect of treatment of subclinical hypothyroidism on children with migraine headache.

**Materials & Methods:**

In a quasi-experimental study, the severity and monthly frequency of headache of 25 migraineur children with subclinical hypothyroidism who were referred to the pediatric neurology clinic of Shahid Sadoughi University of Medical Sciences,Yazd, Iran between January 2010 and February 2011 and were treated with levothyroxine for two months were evaluated.

**Results:**

Thirteen girls (52%) and 12 boys (48%) with the mean age of 10.2 ± 2.76 years were evaluated. In children with hypothyroidism, the monthly frequency of headache (mean ± SD: 17.64 ± 9.49 times vs. 1.2 ± 1.1 times) and the severity of headache (mean± SD: 6.24±1.8 scores vs. 1.33 ± 0.87 scores) were significantly decreased by treatment.

**Conclusion:**

Based on the results of this study, treatment of subclinical hypothyroidism was effective in reducing migraine headaches. Therefore, it is logical to check thyroid function tests in migraineur children.

## Introduction

Headache is one of the most common symptoms in childhood which occurs in more than 90 percent of school-aged children (1). Migraine is the most frequent acute intermittent primary headache in childhood which is characterized by recurrent episodes of headache accompanied by autonomic symptoms such as photophobia, phonophobia, nausea, vomiting, abdominal pain and relief with sleep (2). Its prevalence increases with age and 10.6 percent of 5 to 15-year-old children have migraine headaches (2, 3). Headaches which are related to hypoxia and hypercapnia, dialysis, arterial hypertension, hypothyroidism, fasting and cardiac diseases are classified as metabolic headaches (4). 

Hypothyroidism may exacerbate primary headaches in some of the patients and it can be a risk factor for the occurrence of new daily persistent headache. Based on the second edition of the International Classification of Headache Disorders, it is considered as a headache related to homeostasis (5).

The prevalence of thyroid dysfunction in general population varies from 4.8 to 9 percent (6) and the prevalence of headache in patients with hypothyroidism differs from 14 to 73 percent in different studies (7).

There have been no studies carried out in children and it suggests that the results available from other studies are not generalizable to this age group. The aim of this study was to evaluate the efficacy of levothyroxine on the frequency and severity of migraine headaches of 5 to 15-year-old children with subclinical hypothyroidism.

## Materials & Methods

In a quasi- experimental study, the severity and monthly frequency of headache of 5 to 15-year-old migraineur children with subclinical hypothyroidism who were referred to the pediatric neurology clinic of Shahid Sadoughi University of Medical Sciences,Yazd, Iran between January 2010 and February 2011 and were treated with levothyroxine for two months were evaluated. The sample size of 25 children was assessed based on Z formula and a confidence interval of 95% with 80% power to detect any significant difference between the two groups with a significance level of 0.05. 

The diagnostic criteria for migraine headache in this study were based on the second edition of International Headache Society Classification by taking history and clinical examination (8).

Children with hemiplegic migraine, basilar-type migraine, retinal migraine, childhood periodic syndromes as migraine variants (cyclic vomiting, abdominal migraine and benign paroxysmal vertigo of childhood), children with epilepsy, systemic diseases such as asthma, diabetes mellitus, hepatic and renal diseases, definite secondary headaches, previously diagnosed hypothyroidism or hyperthyroidism and those who had taken drugs that had affected thyroid functions during the past two months were excluded. 

Scoring of the severity of the headache, which was between 0 and 10 points, was done by the patients. Zero represented no pain and 10 points was the most severe pain. Classification of the severity of headache was done as follows: mild, 1-3; moderate, 4-7; and severe, 8-10. After diagnosis of migraine headache in patients based on clinical evaluation, free thyroxin (T4) and thyroid stimulating hormone (TSH) serum levels were measured for evaluating thyroid functions in all of the children. According to Nelson Textbook of Pediatrics, normal values of serum free T4 level at ages 3-10 and in more than 10-year-old children are 5.5 -12.8 and 4.2 -13 microgram per deciliter (μg/dL), respectively. The TSH level is also 0.7-6.4 miliunit per liter (mIU/L) in 5 to 15-year-old children. When the child has no clinical sign of overt hypothyroidism, but the TSH level is more than 4.5 mU/L and either of T4 or free T4 level is normal, it is defined as subclinical hypothyroidism (9).

Serum TSH levels were measured by the third generation of radioimmunoassay methods (chemiluminescent assays) in the reference laboratory of Shahid Sadoughi University of Medical Sciences,Yazd, Iran.

If a patient had a low T4 level, it would be considered as overt hypothyroidism and the child was excluded from the study. Then in the second phase of the study, migraineur children with subclinical hypothyroidism were treated with levothyroxine and were visited for two consecutive months and clinical information regarding the severity and monthly frequency of the headache were recorded through interviewing the patients and finally the patients’ condition were compared before and after treatment. The optimal level for TSH after treatment was considered as 0.4–4 mU/L (9).

The data were analyzed using Statistical Package for the Social Sciences version 15 (SPSS, Chicago, IL, USA). Chi-square test or Fisher exact test was used for data analysis of qualitative variables and the mean values were compared using independent T-test. Differences were considered significant at P values of less than 0.05.

An informed consent was obtained from the parents of migraineur children and this study has been approved by the Ethics Committee of Ali-ebn-Abitaleb School of Medicine, Islamic Azad University, Yazd Branch, Yazd, Iran.

## Results

One hundred four children with migraine headache were evaluated. Based on the interpretation of thyroid function tests, 25 (24%) children including 13 (52%) girls and 12 (48%) boys with the mean age of 10.2 ± 2.76 years had subclinical hypothyroidism.

The mean age onset of migraine was 7.6 ± 1.43 years. Twenty one (84%) children had migraine headache without an aura and the type of aura was visual in two and perioral paresthesia in two of the children.

The severity of headache was mild in two (8%), moderate in 20 (80) and severe in three (12%) children. The most frequent related migraine symptom was nausea (67%). 

The others were vomiting (20%), photophobia (8%) and phonophobia (5%). 

Comparison of the severity, monthly frequency and duration of headache in children with subclinical hypothyroidism before and after levothyroxine treatment is shown in Table I which indicates that monthly frequency and severity of headache significantly decreased with levothyroxine treatment.

Table. II shows the frequency distribution of good response to levothyroxine (all headaches stopped or more than 50 % reduction in the monthly headache frequency) based on sex and migraine type indicating that the drug was more effective in common migraine (without aura) type.

## Discussion

In the present study, efficacy of subclinical hypothyroidism treatment in pediatric migraine headache was evaluated.

In this study, 52% of the patients with migraine headach, were girls and 48% were boys. Based on other studies, the prevalence of migraine was more in boys before the age of seven. It is similar in boys and girls at 7 -11 years of age and is higher in boys up to the age of 14 and is greater in women thenceforward (10- 12). 

In the present study, 24% of migraineur children had subclinical hypothyroidism. In a study conducted by Singh, the association between migraine and hypothyroidism was reported (13) and in a Turkish study, 0.4 and 1.3 percent of 16-69-year-old migraineurs had hypothyroidism and subclinical hypothyroidism, respectively (6).

In a study performed by Moreau et al., 30% of the adult patients with hypothyroidism had headache and 50% of them resolved within two weeks after treatment (14) and in a study in Japan, none of the thirty adult patients with chronic headache had hypothyroidism (15). 

In a study carried out in Norway, in patients aged 20 years and more, high TSH values were associated with a low prevalence of headache, especially in women without a history of thyroid dysfunction, but in people who suffered from headache, especially in migraineurs, TSH was lower than those without headache (7). Tepper et al. reported that in USA, hypothyroidism was more prevalent in patients with new daily persistent headache than in migraineurs and chronic post traumatic headaches (5).

Based on the results of the present study, treatment of subclinical hypothyroidism was effective in reducing migraine headaches and it is logical to check thyroid function tests in children with migraine headache and it is not in agreement with Toprak et al., who concluded that evaluation of thyroid functions is not necessary in adult migraineurs (6). Possible explanations for this discrepancy are differences in the age of the patients and the race. 

Many medical disorders including hypothyroidism are accompanied with or even occur with headache in children (16,17) that should be considered in the evaluation of pediatric headache for early diagnosis and treatment of these treatable causes of primary headache. 

**Table 1:** Comparison of the Severity, Monthly Frequency and Duration of Headache in Children with Subclinical Hypothyroidism Before and After Levothyroxine Treatment

**Group**	**Before treatment**	**After treatment**	**P.value**
**Data**			
Severity of headache in scores (Mean ± SD)	6.24 ± 1.8	1.33 ± 0.87	0.001
Monthly frequency of headache (Mean ± SD)	17.64 ± 9.49	1.2 ± 1.1	0.0001
Duration of headache in hour (Mean ± SD)	1.96 ± 1.08	1.54 ± 1.04	0.09

**Table 2:** Response to Levothyroxine Treatment Based on Sex and Migraine Type

**Good response**		**Yes**	**No**	**P.value**
**Data**				
Sex	Female	9	4	0.66
	Male	10	2	
Migraine Type	Without aura	17	4	0.03
	With aura	2	2	

**In conclusion, **in this study, subclinical hypothyroidism treatment was effective in decreasing the severity and monthly frequency of migraine headaches. Since thyroid diseases may cause headaches and the diagnosis and treatment of hypothyroidism in migraineur children may help and subside prolong migraine prophylactic drugs, it is therefore logical to check thyroid function tests. 
